# GABAergic astrocytes in Alzheimer’s disease

**DOI:** 10.18632/aging.101870

**Published:** 2019-03-15

**Authors:** Olga Garaschuk, Alexei Verkhratsky

**Affiliations:** 1 Institute of Physiology, Department Neurophysiology, Eberhard Karls University of Tübingen, Tübingen, Germany; 2 Faculty of Biology, Medicine and Health, The University of Manchester, Manchester M13 9PT, UK; 3 Center for Basic and Translational Neuroscience, Faculty of Health and Medical Sciences, University of Copenhagen, 2200 Copenhagen, Denmark; 4 Achucarro Center for Neuroscience, IKERBASQUE, Basque Foundation for Science, 48011 Bilbao , Spain

**Keywords:** Alzheimer’s disease, plaque vicinity, neuronal hyperactivity, GABA release, astrocyte

Cellular responsibilities for synaptic transmission in the CNS are neatly divided between neurons, which execute Ca^2+^-regulated, action potential-driven exocytotic secretion of neurotransmitters, and astrocytes that control neurotransmitter clearance and catabolism as well as supply neurons with neurotransmitter precursors [1]. For this purpose astrocytes are endowed with several sets of plasmalemmal transporters (for glutamate, GABA, adenosine, noraderenaline/dopamine, and glutamine) and enzymes (glutamine synthetase, monoamine oxidase B (MAO-B) and adenosine kinase). The glutamate(GABA)-glutamine shuttle in particular is fundamental for principal excitatory (glutamaterogic) and inhibitory (GABAergic) synapses. In healthy mature CNS astrocytes accumulate and process about 80% of synaptically released glutamate. GABA is mostly taken up by neuronal transporters, whereas astrocytes remove ~20% of this inhibitory transmitter. After entering the astrocyte glutamate is normally converted into glutamine by glutamine synthetase whereas GABA is mainly catabolized by GABA transaminase (GABA-T) to succinate, which is subsequently fed into the Krebs cycle and used for energy production. Therefore, in the healthy adult brain of humans and mice cytosolic GABA concentration in astrocytes is low [2–4].

**Figure 1:**
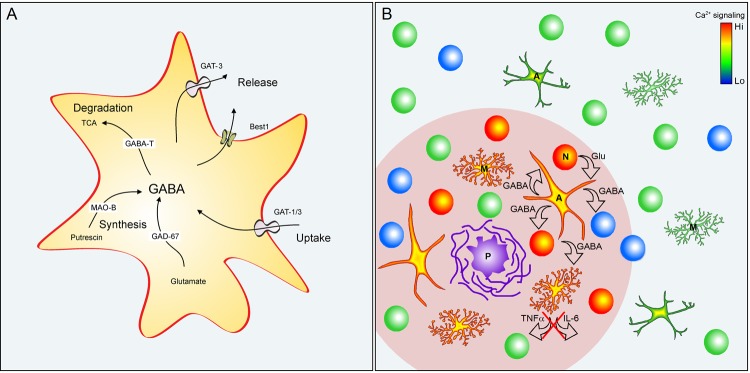
**GABAergic astrocytes in AD.** (**A**) GABA pathways in reactive astrocytes. Abbreviations: GAT1/3 GABA transporters 1 (SLC6A1) and 3 (SLC6A11); Best1 - bestrophin 1 anion channel 1; GABA-T - GABA transaminase; TCA - tricarboxylic acid (Krebs) cycle; MAO-B - Monoamine oxidase B; GAD67 - glutamate decarboxylase. (**B**) Reactive astrocytes in the vicinity of amyloid plaques in AD. Plaque (P) vicinity (pink shading) contains normal (green), silent (blue) and hyperactive (red) neurons (N), as well as hypertrophic astrocytes (A) and microglia (M) with enhanced Ca^2+^ signaling. Glutamate released, for example, from hyperactive neurons is taken up by astrocytes and metabolized to GABA. GABA release (i) inhibits neuronal activity and (ii) release of pro-inflammatory markers (e.g. TNF-α or IL-6) from astrocytes and microglia.

Astroglial GABA content, however, is significantly increased in elderly humans [5], Alzheimer’s disease (AD) patients [2,3] and in amyloid-depositing mouse models of AD (AD mice [2–4]). These transgenic mice carry amyloid precursor protein and presenilin 1 genes with familiar AD mutations, presenting with overt amyloidosis, neuroinflammation, cognitive impairment and characteristic hyperexcitability of cortical and/or hippocampal neuronal networks [6]. Compared to age-matched healthy controls, the cytosolic GABA content of GFAP-positive hypertrophic reactive astrocytes in AD mice increases several times and approaches that of neighboring GABAergic neurons [2–4]. In mice and humans the AD-mediated conversion of astrocytes into GABAergic cells is accompanied by an increased expression of glutamic acid decarboxylase GAD67 and astrocyte-specific GABA transporter GAT3/SLC6A11, whereas in mice an increased expression of astroglial MAO-B and somatic re-location of Ca^2+^-activated, GABA-permeable bestrophin 1 (Best1) channels were also reported [3]. The level of GABA-T remained, however, unchanged [3]. These results highlight two possible pathways for astroglial synthesis and release of GABA (Figure 1A). GABA can be synthesized either from glutamate by GAD67 (likely occurring when expression or activity of glutamine synthetase is decreased as has been shown for reactive astrocytes in AD mouse model, Olabarria et al, Mol. Neurodegener. 2011; 6:55) or from putrescine via a (non-canonical) pathway involving MAO-B. Astroglial release of GABA may potentially occur through diffusion via Best1 channels or through reversal of GAT3 transporters. Consistently, tonic GABA release from astrocytes was documented in two different mouse models of AD (5XFAD and APP/PS1 mice [2,3]). In the cortex and hippocampus of AD mice hypertrophic GABAergic astrocytes have been detected exclusively in the vicinity of amyloid-β plaques [3,4], thus sharing the habitat with hyperactive neurons and hypertrophic microglia with increased Ca^2+^ signaling (Figure 1B). Noteworthy, hypertrophic astrocytes also show pathologically increased Ca^2+^ signaling [6], arguably facilitating GABA release through Ca^2+^-activated Best1 channels.

How do GABAergic astrocytes contribute to AD pathophysiology? By documenting their negative impact on synaptic plasticity in the dentate gyrus as well as on spatial learning and memory in mice, two of the above mentioned studies considered their role as detrimental and proposed that "selective inhibition of astrocytic GABA synthesis or release may serve as an effective therapeutic strategy for treating memory impairment in AD" [2,3]. However, with its extremely sparse neuronal firing dentate gyrus is barely representative of AD-related functional impairment in the forebrain. Indeed, other hippocampal and cortical structures in mice and humans are markedly more liable to AD-related hyperactivity rather than to silencing [6]. Moreover, in the cortex and hippocampus the dependence between amyloidosis and GABA accumulation in astrocytes is bell-shaped, rendering exogenous inhibition of astrocytic GABA synthesis ineffective at later stages of the disease.

Interestingly, transgenic mice harboring only an AD-related presenilin 1 mutation show neuronal hyperactivity and GABA accumulation in astrocytes even without amyloidosis and neuroinflammation [4,7]. It is therefore conceivable that GABA accumulation in astrocytes occurs at the very beginning of the vicious cycle of AD. Therefore we propose that remodeling of reactive astroglia in AD converts them into GABAergic cells capable of GABA release into the brain parenchyma. This GABA release carries two fundamental roles: (i) activation of neuronal GABA_A_ and GABA_B_ receptors with subsequent inhibition of glutamate release, thus palliating neuronal hyperactivity and (ii) suppression of the proinflammatory response of both astrocytes and microglia (Figure 1B). Such anti-inflammatory action of GABA was documented in cultured human astrocytes and microglia. Here GABA reduced the lipopolysaccharide-evoked activation of proinflammatory pathways mediated by NFκB and P38 MAP kinase as well as release of the proinflammatory cytokines TNFα and IL-6 [5]. As a side effect of this homeostatic response astrocytic GABA release may indeed inhibit synapses with low release probability thus causing neuronal silencing in the dentate gyrus or in the vicinity of senile plaques (Figure 1B). The main effect, however, seems to be anti-neurotoxic and anti-inflammatory and thus beneficial. Of note, accumulation of GABA in astrocytes also accompanies tissue injury [3], suggesting that astrocytic GABA-mediated neuroprotection might be a common feature of reactive astrocytes. With the development of the chronic disease, however, astrocytes may convert to senescent instead of reactive phenotype, keeping their hypertrophic appearance but losing the ability to synthesize GABA [4].
